# Hospital Expenditure.—The Commissariat

**Published:** 1896-09-26

**Authors:** 


### The Institutional Workshop.

### HOSPITAL EXPENDITURE.—THE COMMISSARIAT.

### XXVIII.—THE GREENGROCER'S BILL.

There is a great difference in practice among hospitals as to what is provided under the heading of "vegetables." In some institutions potatoes are the only vegetable provided for the patient. In some, carrots, turnips, and onions used in making broth find a place. In others, again, an allowance of "green-meat," cabbage, kale, sprouts, etc., is served two or three times a week. In victualling the rest of the household, even wider variations exist in this respect. Many hospital managers seem to ignore the existence of every vegetable except the potato and the cabbage, and to regard fruit as an entirely needless extravagance, while the clever housekeeper will succeed in providing her nurses with each vegetable in season, and plentiful salads, at a cost no greater than that of the unpopular dish of sodden green leaves common to many institution tables. The same may be said of fruit, for however expensive this may be to provide continuously, almost every kind has its own season of abundance when no pudding is cheaper.

Not many hospitals can draw supplies from their own gardens, nor are prices much cheaper in the country than in London. The largely-increased cost per head of this item in London hospitals must be attributed partly to the existence of a higher standard of living as regards fruit and vegetables, and partly to the fact that it is precisely under this heading that gifts in kind flow most freely into provincial hospitals. The report of many hospitals in country districts show a long list of acknowledgments of hampers of apples, pears, and plums, sacks of potatoes, and many miscellaneous gifts of rhubarb, lettuce, cabbage, etc., and all kinds of fruit and vegetables. The gifts thus contributed might be largely increased by a little energy on the part of secretaries in awaking a local interest in the needs of the hospital, and by a little organisation. Many people with large kitchen gardens habitually lament an avoidable waste in fruit and vegetables, which results partly from over-production and partly from the way things have of ripening all together at one time, and nearly everyone would be better pleased to give their surplus to the hospital than to let it rot on the ground. The only thing that is required is an easy means of transport, for the packing and despatching of hampers is not only troublesome, but expensive. If the hospital authorities would arrange to send a cart, or even a donkey with paniers, for the produce, the cost of conveyance would be far more than recouped, a welcome variety of diet would be secured for the patients and staff, and, not least in importance, an interest in the hospital would be roused and maintained in many new quarters.

The following table shows the annual amount expended on fruit and vegetables, per head, taking all the inmates of the hospital together.

| | £ | s. | d. |
|---|---|---|---|
| St. Thomas's (potatoes only) ... | 0 | 4 | 0 |
| Westminster (potatoes only) ... | 0 | 5 | 0 |
| Great Northern Central | 0 | 10 | 0 |
| Royal Free ... | 0 | 11 | 0 |
| Charing Cross ... / Seamen's ... | 0 | 13 | 0 |
| St. Mary's ... / London ... | 0 | 14 | 0 |
| Middlesex ... | 0 | 15 | 0 |
| University ... | 0 | 17 | 0 |
| St. George's ... | 1 | 0 | 0 |
| Metropolitan ... | 1 | 7 | 0 |

| | £ | s. | d. |
|---|---|---|---|
| Hull Royal ... / York County ... | 0 | 4 | 0 |
| Cambridge Addenbrooke ... | 0 | 5 | 0 |
| Halifax ... | 0 | 8 | 0 |
| Oxford Radcliffe ... | 0 | 9 | 0 |
| Leeds General ... | 0 | 9 | 0 |
| Bristol General ... | 0 | 10 | 0 |
| Birmingham General ... / Birmingham Queen's ... / Sheffield General ... / Norfolk and Norwich ... | 0 | 11 | 0 |
| Newcastle-on-Tyne ... | 0 | 12 | 0 |
| Liverpool Royal... ... | 0 | 13 | 0 |
| Sussex County, Brighton ... | 1 | 0 | 0 |
| Aberdeen... ... | 0 | 4 | 0 |
| Glasgow Royal ... | 0 | 5 | 0 |
| Glasgow Western ... | 0 | 7 | 0 |
| Edinburgh Royal ... | 0 | 8 | 0 |
| Ayr County ... / Belfast Royal ... | 0 | 11 | 0 |

There is little clue to determining what proportion of this must be assigned to the patients, seeing that not only do customs vary, but prices are exceptionally capricious. At Guy's the cost of vegetables works out at 12s. per head for patients, and 24s. for household, but it is evident that less than half this amount must be assigned to the patient in many institutions. If potatoes only are provided, as appears to be the case in many diet tables, about 1 cwt. a year might be reckoned for each occupied bed, the allowance being in all cases ½ lb. weighed uncooked. This reckoning provides for nearly two-thirds of the patients being on meat diet, and about coincides with the actual cost of potatoes alone at St. Thomas's and Westminster. At the Leeds General Infirmary, dividing the quantity of potatoes consumed during the year by the total number of inmates, the yearly quantity consumed per head works out at 146 lbs., or if 1 cwt. be allowed for each occupied bed, then the remainder divided among the members of the household gives 2 cwt. each, which is little more than ½ lb. per head, and may be considered the normal household allowance.

But the following table of prices paid for potatoes in London alone will show that even when the quantity used by the patient is settled the cost is still far from being determined:

| | s. | d. | |
|---|---|---|---|
| Potatoes per bushel | 3 | 0 | and 3s. 4d. |
| " per cwt. ... | 2 | 9 | all round price. |
| " " ... | 3 | 3 | winter; 3s. 9d. summer. |
| " " ... | 3 | 6 | all round price. |
| " " ... | 3 | 9 | " " |
| " " ... | 4 | 0 | " " |

| | s. | d. | |
|---|---|---|---|
| Potatoes, per cwt. | 4 | 3 | all round price. |
| ,, ,, ... | 4 | 6 | ,, ,, (Two hospitals.) |
| ,, ,, ... | 5 | 0 | ,, ,, |
| ,, ,, ... | 5 | 4 | ,, ,, |
| ,, ,, ... | 6 | 0 | ,, ,, |
| ,, ,, ... | 6 | 6 | ,, ,, |
| ,, ,, ... | 5 | 0 | to 10s. (!) varying. |

The interesting point about these prices is the fact that only two hospitals coincide in paying at the same rate for their potatoes. Once again the desirability of a co-operative hospital store is brought forcibly to mind. The wastefulness of bad potatoes is so glaring in their preparation for table, and the unsatisfactory result when cooked is so very notorious to all beholders (not to mention the consumers), that it is hardly likely that even at the cheapest of the above quotations a distinctly inferior article is supplied. It is more probable that in some hospitals a cheap source of supply from the country has been discovered, and it is exactly this office of buying in the cheapest market, which should be undertaken for all hospitals by a central distributing store. The high prices at the close of the list can only be accounted for by ignorance of the market.

Outside London prices rule very variously. At Birmingham and Hull, 3s. per cwt.; at Norwich, 3s. 5¼d.; at Sheffield, 3s. 6¾d.; at Newcastle-on-Tyne, 3s. 9¾d.; at Leeds, 4s. 10½d.; at Derby, 5s. In Scotland the price rises from 3s. per cwt. at Edinburgh and Aberdeen, to 3s. 4d. and 4s. at Glasgow, and to 6s. per cwt. at Ayr.

Deducting for each occupied bed a sum of from 3s. to 6s. for potatoes, there remains in some provincial institutions less than ¼d. a day, or 7s. 6d. a year, each, to provide fruit and vegetables for the other inmates. This sum is entirely inexplicable, except on the supposition before-mentioned of gifts in kind, and can hardly even then be accepted as a fair average for a well-arranged dietary. Fruit at least twice a week, and some kind of vegetable daily in addition to the ½lb. of potatoes should form an invariable part of every household bill of fare. It is hardly possible to provide this under ½d. per day each, or 15s. a year, and it does not appear that the gifts of fruit and vegetables in any hospital are sufficient, when the staff is at all numerous, to make up the deficiency we have noted.

